# Toothpick: An Unusual Cause of Small Bowel Perforation in an Adult

**DOI:** 10.7759/cureus.43008

**Published:** 2023-08-05

**Authors:** Klara Schwarzova, Robert J Dabek, Aubrey Mwinyogle, Gerald Hayward

**Affiliations:** 1 Surgery, Ascension St. Agnes Hospital, Baltimore, USA

**Keywords:** toothpick, laparotomy decision, bowel perforation, foreign body ingestion, small bowel obstruction

## Abstract

Foreign body ingestion is a common complaint in the pediatric population; however, in adults, this entity remains quite rare. Most cases are managed conservatively with serial examinations and imaging. Rarely, foreign body ingestion may cause small bowel perforation and peritonitis in adults. Perforation often warrants operative management, and assessment of bowel viability is crucial. Here, we present a case of foreign body ingestion requiring exploration, without the need for bowel resection or repair. Although the need for operative intervention in adults after foreign body ingestion remains rare, it is crucial to recognize those patients who are both at risk for foreign body ingestion and have underlying small bowel narrowing that puts them at risk for perforation. A high index of suspicion in these instances is mandatory as early recognition and appropriate treatment will improve outcomes.

## Introduction

Foreign body ingestion is a common complaint in the pediatric population; however, in adults, this entity remains quite rare. Several risk factors for unintentional foreign body ingestions in adults have been identified. These include psychiatric illness, cognitive impairment, dentures, and advanced age [1-3]. The majority of patients can be safely observed and monitored with serial imaging. In about 20% of patients, endoscopic removal is warranted, and less than 1% require operative intervention [2]. We present an interesting case of a contained small bowel perforation in an adult that required an exploratory laparotomy and retrieval of the foreign body.

## Case presentation

**Figure 1:**
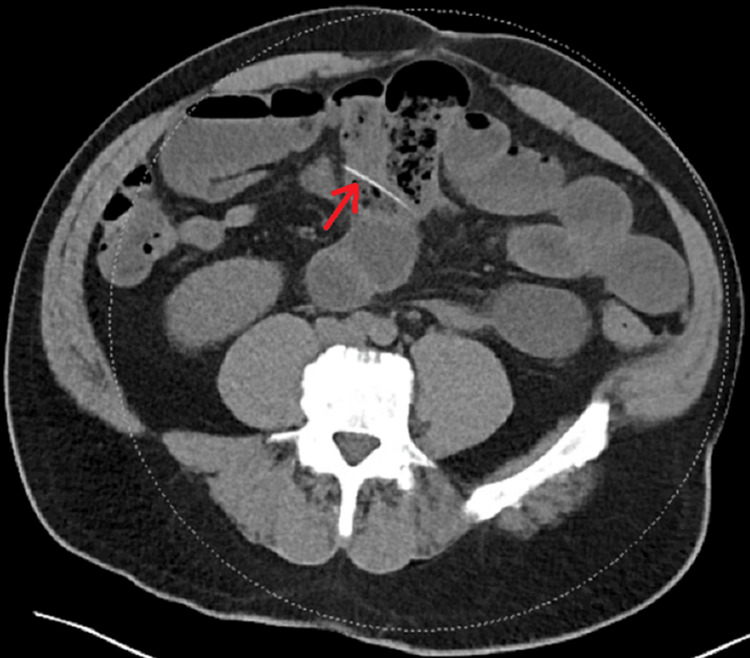
Axial non-contrast CT imaging of the abdomen and pelvis demonstrating the linear hyperlucent object appearing to lie within a loop of the small bowel.

A 59-year-old male with no significant past medical history and a past surgical history of trauma laparotomy 15 years prior presented to the emergency room with abdominal pain, nausea, and vomiting of two days duration. His last meal prior to the presentation was a chicken sandwich. Of note, the patient wears dentures at baseline. At presentation, physical examination was notable for abdominal distention and mild diffuse tenderness to palpation without peritoneal signs. Laboratory workup showed a marginally elevated white blood cell (WBC) count of 10,600 per microliter (normal range 4,000-11,000) and lactic acid of 1.6 mmol/L. Computed tomography (CT) scan of the abdomen and pelvis (Figure 1) showed dilated loops of the small bowel with a taper towards a bowel loop containing a linear radiopaque object causing a partial bowel obstruction. At that time, our differential included bowel obstruction due to adhesions, or due to the foreign body, or ileus. We hypothesized that the foreign object was either a chicken bone, given the patient's history, or a needle, given the radiopaque appearance on CT. The patient was initially managed with nasogastric tube (NGT) decompression and serial abdominal examination; however, the following morning, his abdominal exam was concerning for peritonitis. Therefore, he was taken to the operating room for exploration.

**Figure 2:**
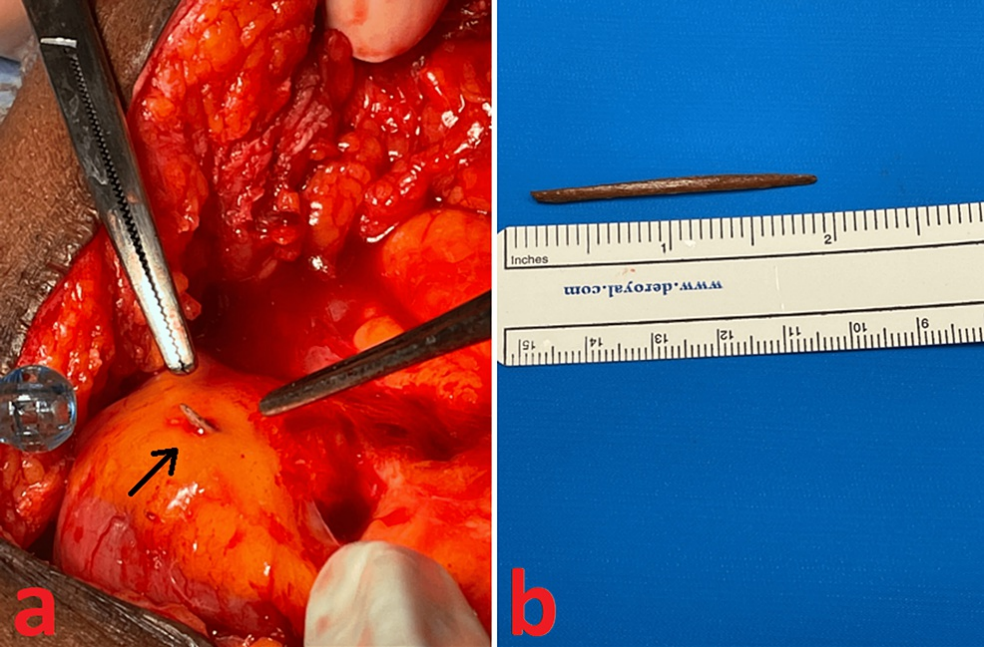
Intraoperative and gross pathology photos (a) Small bowel mesentery with a foreign object as discovered intraoperatively, indicated by black arrow. (b) Gross examination of the removed specimen, measuring approximately 4.6 cm.

Upon entering the peritoneal cavity, there were several adhesions between the loops of the small intestine. However, none of them were clearly causing an obstruction. The small bowel appeared injected, which would be consistent with mild serositis; however, there was no purulence or fibrinous material encountered. The bowel was examined from the ligament of Treitz to the ileocecal valve. In the dilated ileum, a 4.6-cm linear object was identified in the small bowel mesentery fat (Figures 2a, 2b). The object was extracted after cautious sharp dissection within the mesenteric fat. A saline submersion test was negative for perforation, suggesting that the object pierced the small bowel wall and migrated into the mesentery while the exit point had already been sealed. Given the affected loop of the bowel appeared viable and was contracting, a decision was made not to perform a small bowel resection after the foreign object was removed. Postoperatively, the patient recovered well and was discharged home on postoperative day 5, tolerating a soft diet. The pathology of the object was consistent with a plant-based material, most likely a toothpick. At a two-week follow-up, he was recovering well and had no complaints related to his surgery. A review of regional medical records at one-year postoperatively did not show evidence of any further obstructive symptoms or complications.

## Discussion

Foreign body ingestion is an uncommon cause of small bowel perforation and peritonitis in adults. A combination of risk factors, such as advanced age, dentures, and clinical signs of bowel perforation, should prompt appropriate investigation. In our case, we suspected that the patient's recent history of eating a sandwich was merely a coincidence and that the foreign object was swallowed a while ago. The treating physician must keep in mind that although rare, foreign body ingestion might be the cause of perforation and peritonitis. Perforation frequently occurs in areas of narrowing, whether physiologic, such as at the ileocecal valve, or pathologic caused by strictures in the setting of adhesions, Crohn's disease, or small bowel tumors [1,2]. Ingested objects that are more likely to cause perforation are sharp objects such as fish or chicken bones, needles, and toothpicks [1]. In general, conservative management is appropriate for the majority of ingestions, since most objects will pass through the GI tract uneventfully [4-6]. Objects found to be retained in the esophagus should be emergently removed within 24 hours. Patients with known sharp objects, objects > 5 cm in length, or magnets should be removed promptly if they are accessible for endoscopic retrieval [4-6].

Investigation poses a challenge in cases where the ingested object is unknown, radiopaque, or not large enough to be seen on imaging. Plain abdominal x-ray films are oftentimes not sensitive enough to show thin objects such as chicken or fish bones [7]. On the other hand, CT can have up to 100% sensitivity for the identification of foreign bodies, though, in some instances, objects were identified retrospectively on CT after intraoperative discovery [1]. In our case, the CT was able to delineate a toothpick made from plant/wooden material, as shown in the final pathology report. The object was roughly 5 cm, however, was distal enough from the stomach that endoscopic retrieval would be unfeasible. Then, with the development of peritoneal signs, conservative management was no longer appropriate.

In regard to intraoperative management, assessment of bowel viability is crucial. Segmental bowel resection may be warranted in cases of free intraperitoneal perforation [1]; however, in our case, there was an absence of peritoneal contamination and lack of a bowel wall perforation, and the affected small bowel mesentery appeared intact - we thus opted for bowel preservation and closed the abdomen after washout.

## Conclusions

The need for operative intervention in adults after foreign body ingestion remains rare. However, it is crucial to recognize those patients who are both at risk for foreign body ingestion and have underlying small bowel narrowing that puts them at risk for perforation. A serial abdominal examination would be appropriate in the absence of peritoneal signs. A high index of suspicion in these instances is mandatory as early recognition and appropriate treatment will improve outcomes.
